# Integrated Care in Patients with Atrial Fibrillation and Optimal Medical Treatment for Heart Failure: Results from the Heart failuRe ObsErvational Study (HEROES)

**DOI:** 10.3390/jcm14238338

**Published:** 2025-11-24

**Authors:** Iwona Gorczyca-Głowacka, Karolina Molenda, Maciej Nadel, Agata Galas, Agata Tymińska, Katarzyna Byczkowska, Aleksander Siniarski, Witold Furmanek, Adrian Stefański, Beata Wożakowska-Kapłon, Dominika Klimczak-Tomaniak, Robert Morawiec

**Affiliations:** 1 Collegium Medicum, The Jan Kochanowski University, 25-317 Kielce, Poland; bw.kaplon@poczta.onet.pl; 2 Department of Psychiatry, Swietokrzyskie Psychiatry Center, 26-026 Morawica, Poland; xkarolinamolenda@gmail.com; 3 2nd Department of Cardiology, Medical University of Lodz, 90-549 Lodz, Poland; maciej.nadel@umed.lodz.pl (M.N.); robert.morawiec@umed.lodz.pl (R.M.); 4 Department of Cardiology and Internal Diseases, Military Institute of Medicine, 04-349 Warsaw, Poland; agalas@wim.mil.pl; 5 First Department of Cardiology, Medical University of Warsaw, 02-097 Warsaw, Poland; tyminska.agata@gmail.com; 6 Department of Heart Failure, Transplantology and Mechanical Circulatory Support, National Institute of Cardiology, Alpejska 42, 04-628 Warsaw, Poland; kbyczkowska@ikard.pl; 7 Department of Coronary Disease and Heart Failure, Institute of Cardiology, Jagiellonian University Medical College, 31-202 Krakow, Poland; aleksandersiniarski@gmail.com; 8 Institute of Heart Diseases, Wroclaw Medical University, 50-566 Wroclaw, Poland; witold.furmanek@gmail.com; 9 Department of Hypertension and Diabetology, Faculty of Medicine, Medical University of Gdansk, 80-214 Gdansk, Poland; adrian.stefanski@gumed.edu.pl; 10 1st Clinic of Cardiology and Electrotherapy, Swietokrzyskie Cardiology Center, 25-736 Kielce, Poland; 11 Department of Cardiology, Hypertension and Internal Medicine, Medical University of Warsaw, 03-258 Warsaw, Poland; dominika.klimczak@wum.edu.pl

**Keywords:** atrial fibrillation, heart failure, integrated care, optimal medical treatment

## Abstract

**Background:** Heart failure (HF) and atrial fibrillation (AF) often coexist, and this has a major impact on prognosis. Only a limited amount of data is available on the effect of integrated strategies to improve prognosis in patients with HF and AF. We aimed to evaluate the association between HF subtype, HF optimal medical therapy (OMT), adherence to the Atrial Fibrillation Better Care (ABC) pathway, and all-cause death. **Methods:** Hospitalized patients and outpatients were enrolled in the Heart failuRe ObsErvational Study, which is a prospective, multicenter cohort study, between April 2022 and January 2024. Our analysis included patients with both HF and AF, who were divided into three groups: ABC + OMT, noABC + OMT, and noABC + noOMT. **Results:** A total of 627 patients, of whom 51.7% had HF with reduced ejection fraction, were included in this study. Direct oral anticoagulants were prescribed to 70.8% of patients, and 71.6% of these received the recommended doses. The patients were divided into the following groups: 49.8% were assigned to the ABC + OMT group, 25.2% to the noABC + OMT group, and 25% to the noABC + noOMT group. All-cause death occurred in 19.5% of patients. OMT compliance was associated with lower risk of all-cause death (HR0.61, 95% CI 0.42–0.9, *p* = 0.01), whereas management compliant with the ABC pathway was not (HR0.74, 95% CI 0.51–1.09, *p* = 0.12). In the ABC + OMT group, the risk of all-cause death was lower than in the noABC + noOMT group (HR0.60, 95% CI 0.39–0.92, *p* = 0.02). **Conclusions:** Integrated treatment strategies were implemented in half of the population with HF and AF, and this was associated with better long-term prognosis compared with patients who did not receive a complex treatment strategy.

## 1. Introduction

Heart failure (HF) commonly occurs in patients with atrial fibrillation (AF), and vice versa [1,2]. From a pathophysiological standpoint, these two conditions are interconnected, influencing each other in both directions and sharing common risk factors. This reciprocal relationship creates a vicious cycle, where AF can exacerbate HF and HF can, in turn, worsen AF. AF has been identified as both a cause and a consequence of HF. Both disorders are associated with advancing age and linked to numerous comorbidities, which contribute to their frequent coexistence in individual patients. Additionally, the incidence of both AF and HF is rising worldwide, a trend which is likely to persist as the population ages and life expectancy increases. Therefore, patients with both AF and HF have a much higher risk of adverse clinical outcomes compared with those with only one of these conditions [3,4,5,6,7].

Given the clinical complexity of this patient population, implementing an intensive and structured therapeutic management pathway is one potential approach to improving the prognosis of patients with AF and HF. Patients with HF frequently present with multiple comorbidities and thus require integrated care, with guideline-directed optimal medical therapy (OMT) serving as the management foundation [8].

The Atrial Fibrillation Better Care (ABC) pathway was introduced to facilitate and coordinate comprehensive care for individuals with AF [9]. Adoption of this strategy has been linked to a reduced risk of major adverse outcomes, leading to its incorporation into international guidelines for AF management [10,11,12]. The ABC pathway consists of three core components: “A”, avoidance of stroke via appropriate anticoagulation; “B”, better symptom control through rate or rhythm management; and “C”, cardiovascular and comorbidity risk optimization. In patients with both AF and HF, adherence to the ABC pathway is intrinsically linked to compliance with HF OMT, particularly due to the critical role it plays in fulfilling the “C” component.

Although there is a close association between AF and HF, and a complex treatment strategy is needed for both, comprehensive data in this area are lacking. Therefore, we performed a comparative assessment of the prevalence of integrated treatment strategies, including adherence to OMT and the ABC pathway, and their impact on all-cause mortality in patients with HF and AF.

## 2. Materials and Methods

### 2.1. Study Population

The HEart failuRe ObsErvational Study (HEROES) is a multicenter, prospective, observational registry organized by the Polish Society of Cardiology. From April 2022 to January 2024, consecutive patients with HF—both hospitalized and outpatients—were recruited from 41 centers. Detailed descriptions of the study methodology, patient demographics, and follow-up protocols have been provided in prior publications [13].

Individuals who did not have AF, those with missing left ventricular ejection fraction (LVEF) data, and patients who died during their hospital stay were excluded from the analysis.

### 2.2. Covariates

We collected all included patients’ baseline demographics, medical history, concomitant diseases, diagnostic test results, and pharmacology.

Data regarding the presence of AF were obtained either from patient histories recorded in the HEROES registry or from electrocardiogram findings documented within the study.

In accordance with the current HF classification, patients with a left ventricular ejection fraction (EF) below 40% were categorized as having HF with reduced ejection fraction (HFrEF). Those with an EF between 40% and 49% were classified as having HF with mildly reduced ejection fraction (HFmrEF), whereas an EF of 50% or higher was defined as HF with preserved ejection fraction (HFpEF).

The estimated glomerular filtration rate (eGFR), calculated using the Cockcroft–Gault equation, was used to assess patients’ kidney function.

Thromboembolic risk was defined using the CHA2DS2-VASc score, which incorporates HF, hypertension, age ≥ 75 years, diabetes mellitus, stroke/transient ischemic attack, vascular disease, age 65–74 years, and sex category, in accordance with the European Society of Cardiology 2020 AF management guidelines, applicable throughout the study period.

Anticoagulant therapy was evaluated in the study cohort. The present analysis included patients with both AF and HF who were receiving DOACs and for whom follow-up data were available. Anticoagulant treatment was assessed either at hospital discharge or, for outpatients, following any changes made during clinic visits.

### 2.3. Atrial Fibrillation Better Care (ABC) Pathway Evaluation

According to its original definition, the ABC pathway was assessed as follows:

“A” Criterion: A patient met this criterion if they were appropriately prescribed and treated with an OAC based on thromboembolic risk. Treatment with a vitamin K antagonist (VKA) or a direct oral anticoagulant (DOAC) was considered optimal in male patients with a CHA2DS2-VASc ≥ 1 or female patients with a CHA2DS2-VASc ≥ 2. Patients who did not receive an OAC who were at low thromboembolic risk (CHA2DS2-VASc score of 0 in men or 1 in women) also fulfilled the “A” criterion.

“B” Criterion: Any patient without AF symptoms, or with only mild symptoms not affecting daily life, met this criterion. The evaluation of the “B” component was carried out based on the assessment of the patient’s symptoms as well as on the evaluation of rate or rhythm control. Researchers had data such as a full patient history, medical history information, heart rate, and ECG—for hospitalized patients at discharge and admission.

“C” Criterion: To evaluate adherence to the “C” criterion, the most frequent comorbidities associated with AF were considered: hypertension, coronary artery disease, peripheral artery disease, HF, stroke/transient ischemic attack, and diabetes mellitus. A patient qualified for the “C” criterion if they had at least one of these conditions and received the best medical therapy according to the current clinical guidelines. Optimal medical treatment was defined as follows:

- For hypertension: Controlled blood pressure (≤140/90 mmHg) recorded at baseline;
- For coronary artery disease: Treatment with angiotensin-converting enzyme inhibitors, beta-blockers, and statins;
- For peripheral artery disease: Treatment with statins;
- For previous stroke/transient ischemic attack: Treatment with statins;
- For HF: Treatment according to the guideline recommendations for the specific HF subtype [9].

Patients who met all three criteria were considered adherent to the ABC pathway; otherwise, they were classified as ABC nonadherent [9].

### 2.4. Optimal Medical Treatment for Heart Failure

OMT for HF was defined as the comprehensive and contemporary use of drug classes according to HF subtype in concordance with the European Society of Cardiology 2021 HF management guidelines and with the European Society of Cardiology 2023 Update of the previous guidelines, which were valid throughout the study period [8,14]. These therapies were analyzed in relation to LVEF according to the current HF classification: HFrEF, HFmrEF, and HFpEF.

### 2.5. Study Design

Patients were divided into three groups based on adherence to treatment recommendations, specifically adherence to the ABC pathway and compliance with OMT. Group assignment was determined through an analysis of real-life treatment data collected after patient enrollment during the data analysis phase:

- ABC + OMT group: Patients with ABC pathway adherence and with OMT for HF;
- noABC + OMT group: Patients without ABC pathway adherence and with OMT for HF;
- noABC + noOMT group: Patients without ABC pathway adherence and without OMT for HF.

### 2.6. Statistical Analyses

Statistical analyses were conducted using a significance level of α = 0.05. Baseline characteristics were summarized using descriptive statistics. Continuous variables were reported as medians with interquartile ranges (IQRs) due to non-normal distributions, assessed using Shapiro–Wilk tests. Categorical variables were expressed as counts and percentages. Comparisons were made across groups (e.g., ABC + OMT, noABC + OMT, noABC + noOMT) using the Kruskal–Wallis test for continuous variables and the chi-square test or Fisher’s exact test (when expected cell counts were <5) for categorical variables. Post hoc pairwise comparisons were made using the Bonferroni correction, with an adjusted significance level to account for three group comparisons, and significant differences were denoted using the compact letter display (CLD) convention.

Adherence to the ABC pathway and OMT was calculated as the proportion of patients who met the predefined criteria within each HF subgroup (HFpEF, HFmrEF, HFrEF), and this was compared using chi-square tests. Survival analyses were performed to assess all-cause mortality across LVEF categories and treatment groups. Overall survival was visualized using Kaplan–Meier survival curves, and differences in survival probabilities were evaluated using the log-rank test.

Multivariable Cox proportional hazards regression models were used to estimate hazard ratios (HRs) and 95% confidence intervals (CIs) for all-cause mortality. Adjustments were made for potential confounders, including age, sex, body mass index, hypertension, and diabetes mellitus. These variables were selected a priori based on their clinical relevance and established influence on mortality in patients with HF and AF, as supported by prior epidemiological and registry studies. LVEF categories (HFpEF, HFmrEF, HFrEF) and detailed LVEF ranges were analyzed separately, with HFpEF or the lowest LVEF range (10–19%) as the reference groups, respectively. The proportional hazards assumption was verified using Schoenfeld residuals. Similarly, Cox regression was applied to assess the independent and combined effects of ABC pathway adherence and OMT compliance, with noABC + noOMT as the reference group. Interactions between HF subgroups and treatment status were explored by including interaction terms in the models.

Sensitivity analyses were performed to evaluate the robustness of the findings, including Kaplan–Meier analyses stratified by combined ABC and OMT adherence status. In the case of missing data, we restricted the analyses to patients with complete data for specific variables, with sample sizes reported accordingly.

Analyses were conducted using R statistical language (version 4.3.3; R Core Team, 2024). Artificial intelligence was not used in the preparation of this manuscript.

## 3. Results

### 3.1. Patient Characteristics

**Figure 1:**
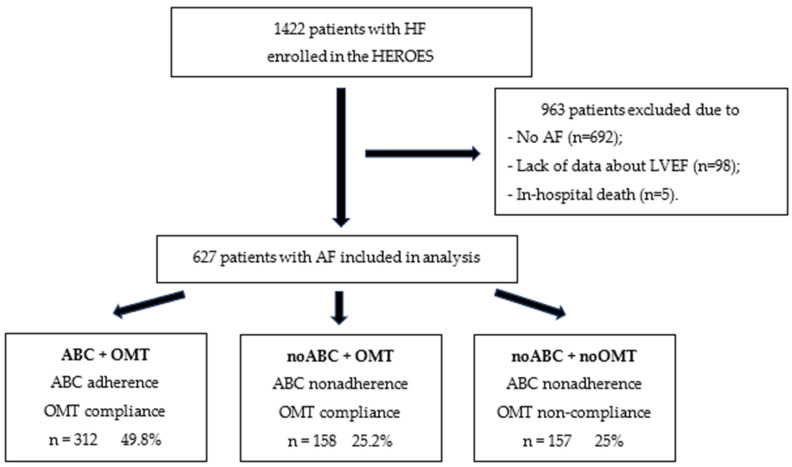
Flowchart of the research process of this study. Abbreviations: ABC—Atrial Fibrillation Better Care pathway; AF—atrial fibrillation; LVEF—left ventricular ejection fraction; OMT—optimal medical therapy.

A total of 1422 patients who provided informed consent were enrolled in the HEROES. A flow diagram illustrating the study process is shown in Figure 1. We excluded 692 patients without AF, 96 patients with unknown LVEF, and 5 patients who died in hospital from the analysis. The total study cohort consisted of 627 patients; of these, 173 (27.6%) had HFpEF, 130 (20.7%) had HFmrEF, and 324 (51.7%) had HFrEF.

The mean age of the overall study population was 70.42 ± 11.49 years; 206 females (32.9%) were enrolled, and 95 patients (20.7%) were enrolled as outpatients.

Among the 627 patients included in the analysis, 557 (88.8%) were treated with OACs, 134 (21.4%) with antiplatelets agents, 133 (21.2%) with angiotensin receptor–neprilysin inhibitors, 329 (52.5%) with angiotensin-converting enzyme inhibitors, 69 (11%) with angiotensin receptor blockers, 583 (92.9%) with beta-blockers, 432 (68.9%) with mineralocorticoid receptor antagonists, and 423 (67.5%) with sodium–glucose cotransporter 2 inhibitors.

### 3.2. Comparison of Patients Across ABC Pathway Adherence and OMT Compliance

Of the overall study population, 312 patients (49.8%) were included in the ABC + OMT group, 158 patients (25.2%) were included in the noABC + OMT group, and 157 patients (25%) were included in the noABC + no OMT group. The patients in the noABC + noOMT group were the oldest. Among HFrEF patients, the proportion in the ABC + OMT group was the highest. A comparison of the patients regarding ABC pathway and OMT adherence is shown in Table 1.

### 3.3. Adherence to the ABC Pathway

Among the patients included in the analysis, 312 patients (49.8%) met all three ABC criteria and were therefore fully managed with ABC-adherent care. In contrast, 18 patients (2.9%) did not meet any of the ABC pathway criteria. Furthermore, 73 patients (11.6%) met only one ABC pathway criterion, while 224 (35.7%) patients met two ABC criteria.

In the study group, 557 (88.8%), 513 (81.8%), and 387 (61.7%) patients were adherent to the A, B, and C criteria, respectively. The prevalence of ABC pathway adherence was observed in 67 patients (38.7%) with HFpEF, in 69 patients (53.1%) with HFmrEF, and in 176 patients (54.3%) with HFrEF (*p* = 0.003).

DOACs were prescribed to 444 patients (70.8%) across the whole study group. Among the DOAC-treated population, 318 patients (71.6%) received the recommended doses, 84 patients (18.9%) received less than the recommended doses, and 42 patients (9.5%) received more than the recommended doses.

### 3.4. Adherence to the OMT

OMT compliance was observed in 470 patients (78.1%). The prevalence of OMT compliance was noted in 244 patients (75.3%) with HFrEF, 102 patients (78.5%) with HFmrEF, and 124 patients (71.7%) with HFpEF (*p* = 0.69). Table 2 outlines details according to ABC pathway adherence among patients with and without OMT compliance.

### 3.5. Outcome According to Spectrum of Left Ventricular Ejection Fraction

**Figure 2:**
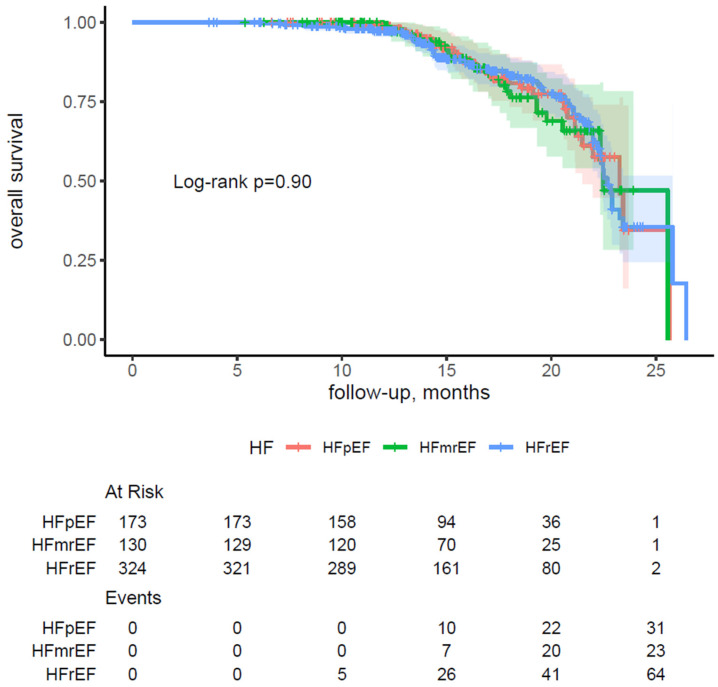
Kaplan–Meier curves for overall survival stratified by heart failure subtypes in patients with atrial fibrillation. Abbreviations: HFmrEF—Heart Failure with Mid-Range Ejection Fraction; HFpEF—Heart Failure with Preserved Ejection Fraction; HFrEF—Heart Failure with Reduced Ejection Fraction.

During a median follow-up period of 15.34 months (IQR: 12.57, 19.37), a total of 122 all-cause deaths (19.5%) occurred in the study cohort. All-cause mortality rates according to HF subtype were as follows: HFpEF vs. HFmrEF vs. HFrEF, 18.50% vs. 18.46% vs. 20.37%, *p* = 0.84. Figure 2 outlines the Kaplan–Meier survival curves for all-cause mortality according to HF subtype.

The log-rank test indicated no significant differences in overall survival among the heart failure subgroups (*p* = 0.90). These findings remained robust in the multivariable analysis. With HFpEF serving as the reference group, neither HFmrEF (HR 1.17, 95% CI 0.68–2.01, *p* = 0.57) nor HFrEF (HR 1.29, 95% CI 0.80–2.09, *p* = 0.30) was associated with a significantly elevated risk of all-cause mortality. Likewise, when evaluating left ventricular ejection fraction as a categorical variable, no significant associations with all-cause mortality were identified across any LVEF category relative to the reference group (LVEF 10–19%), as presented in Table S1.

### 3.6. Impact of ABC Pathway Adherence and OMT on Outcome, Including Interaction and Stratified Analyses

In patients with HF and AF, a lower rate of all-cause death was observed among those who received OMT than among those who did not (16.8% vs. 27.4%, *p* = 0.004). Similarly, patients adherent to the ABC pathway exhibited a lower rate of all-cause death compared with non-adherent patients (15.7% vs. 23.2%, *p* = 0.02).

Multivariable Cox proportional hazards regression analysis, adjusted for confounders including age, sex, body mass index, hypertension, and diabetes mellitus, demonstrated that compliance with OMT was independently associated with a significant 39% reduction in the risk of all-cause mortality (HR 0.61, 95% confidence interval [CI] 0.42–0.90, *p* = 0.01). This protective effect was particularly pronounced in patients with HFrEF, where OMT compliance was associated with a 38% risk reduction compared with no OMT (HR 0.62, 95% CI 0.39–0.98, *p* = 0.04), and showed a borderline significant association in HFpEF (HR 0.58, 95% CI 0.34–1.00, *p* = 0.05), but not in HFmrEF (HR 0.65, 95% CI 0.36–1.15, *p* = 0.14).

In contrast, adherence to the ABC pathway alone was not significantly associated with a reduced risk of all-cause mortality overall (HR 0.74, 95% CI 0.51–1.09, *p* = 0.12) or across HF subtypes, with no significant associations in HFpEF (HR 0.55, 95% CI 0.26–1.17, *p* = 0.12), HFmrEF (HR 1.04, 95% CI 0.59–1.83, *p* = 0.90), or HFrEF (HR 0.70, 95% CI 0.43–1.13, *p* = 0.15).

The combined approach of ABC pathway adherence and OMT compliance was linked to a robust 40% reduction in mortality risk compared with non-adherence to both (HR 0.60, 95% CI 0.39–0.92, *p* = 0.02), whereas non-adherence to the ABC pathway with OMT compliance showed a non-significant trend toward benefit (HR 0.64, 95% CI 0.40–1.02, *p* = 0.06), as detailed in Table S1.

Interaction analyses between ABC pathway adherence and OMT compliance indicated no statistically significant interactions overall (*p* for interaction = 0.45) or when stratified by HF subtypes (*p* for interaction in HFpEF = 0.52, HFmrEF = 0.68, HFrEF = 0.37).

**Figure 3:**
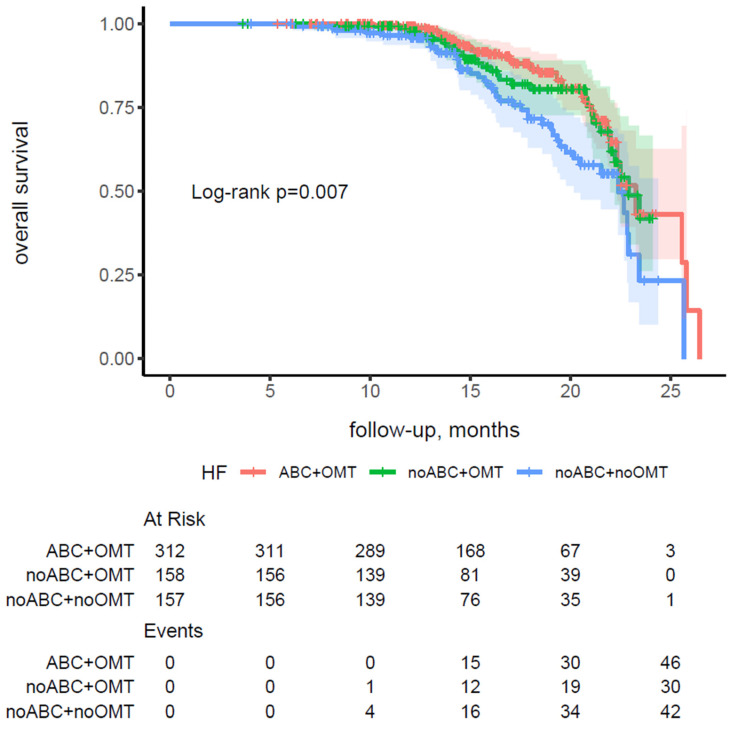
Kaplan–Meier curves for overall survival stratified by adherence to the atrial fibrillation better care pathway and optimal medical therapy in patients with heart failure and atrial fibrillation. Abbreviations: ABC—Atrial Fibrillation Better Care; HF—heart failure; OMT—optimal medical therapy.

These findings were robust in sensitivity analyses and were corroborated by Kaplan–Meier survival curves (Figure 3), which demonstrated a significantly lower cumulative incidence of all-cause death in the ABC + OMT group (log-rank *p* = 0.007).

## 4. Discussion

In this analysis of a large national HF study, the main findings were as follows: Firstly, integrated treatment strategies were implemented in half of the patients with both AF and HF. Secondly, adherence to the ABC pathway varied by HF subtype, whereas adherence to OMT did not differ across HF subtypes. Thirdly, in patients with both AF and HF, adherence to either OMT or the ABC pathway was associated with a reduced risk of all-cause death, with the magnitude of risk reduction being greater for OMT compliance.

Patients with HF, regardless of LVEF, have high mortality and a high risk of hospitalization; moreover, their quality of life is significantly worse compared with patients without HF [8,14,15,16]. Therefore, the use of drugs that positively influence these outcomes remains a cornerstone of long-term therapy for patients with HF. The clinical scenario becomes even more complex when a patient with HF also develops AF. In this group, a multifaceted management approach—including the prevention of thromboembolic complications, symptom control, and the treatment of comorbid conditions (the ABC pathway)—significantly improves prognosis [17,18].

Our study is one of only a few published analyses (the first in the Polish population) that comprehensively assess the effect of HF-specific optimal management (as defined by guideline-directed OMT) and the ABC pathway on the risk of all-cause death in patients with both AF and HF. We found that while the combination of OMT and the ABC pathway is effective in reducing the risk of all-cause death, when considering the impact of OMT and the ABC pathway separately, OMT had a greater effect. This may be due to the fact that OMT in patients with HF, by definition, involves the use of multiple pleiotropic drugs with a broad spectrum of action and a proven effect on improving prognosis. It should also be noted that the lack of OMT compliance in patients with AF and HF is comparable to non-adherence to the ABC pathway.

In contrast, Bonini et al. [19] demonstrated that in a population of patients with AF and HF, the ABC pathway had a greater impact on reducing the risk of adverse outcomes compared with OMT. However, the results of the aforementioned study are consistent with ours regarding the assessment of the effect of integrated management in this group of patients [19].

In our study, integrated treatment strategies were implemented in half of the patients with both HF and AF. While these results are not fully satisfactory, they do indicate that guideline implementation is becoming increasingly comprehensive in clinical practice. When the implementation of management guidelines was considered separately for patients with AF and HF, differences were observed in the application of the ABC pathway and OMT. We noticed that adherence to specific components of the integrated approach to AF management was relatively high (89% of patients with A-adherent management, 82% with B-adherent management, and 62% with C-adherent management). However, adherence to all components of the ABC holistic approach was observed in only half of the patients and thus remained suboptimal. Moreover, the prevalence of ABC pathway adherence was highest among patients with HFrEF. Kozieł et al. [20] showed that in the BALKAN-AF survey’s population, adherence to the ABC pathway was similar to that in our study, at 43.8%. In contrast, data from the BALKAN-AF survey showed a lower rate of patients with A-adherent management. This may be due to the fact that Kozieł et al.’s study [20] was conducted several years earlier than ours, and therefore a lower proportion of patients received appropriate stroke prophylaxis. Particular emphasis is placed on the impact of A-adherent management on prognosis in AF patients. Mitręga et al. [21] showed that patients with AF who received OACs had an almost 5-fold lower risk of death compared with those who were not receiving anticoagulation. In the present study, nearly 90% of patients received A-adherent management, and it was shown that integrated implementation of the ABC pathway and OMT compliance reduces the risk of all-cause mortality. In a large contemporary cohort of European AF patients, clinical management adherent to the ABC pathway for integrated care was even lower than in the studies discussed, at only 30% [22].

In the present study, OMT compliance was observed in 78% of patients. In contrast to ABC pathway adherence, the prevalence of OMT compliance was consistent across all HF subtypes. In recent years, there has been a real breakthrough in the treatment of HF with the introduction of angiotensin receptor–neprilysin inhibitor (ARNI) and sodium–glucose cotransporter 2 inhibitors (SGLT2is), which should be added to the β-blocker and mineralocorticoid receptor antagonist (MRA) therapies that have been used for years, particularly in patients with HFrEF. According to Vaduganathan et al.’s meta-analysis [23], the combination of an ARNI, an SGLT2i, an MRA, and beta-blockers can provide up to 8.3 years of cardiovascular survival and up to 6.3 years of overall survival compared with conventional ACEI plus a β-blocker treatment, constituting a therapy based on complementary pathophysiological mechanisms and the strongest available evidence, with a notable impact on prognosis. Similarly, the SwedeHF study showed that modern HF therapy was associated with a significant 28% reduction in all-cause mortality and a 62% reduction in cardiovascular mortality compared with conventional HF treatment [24]. Associations with effects on outcomes were consistent across all subgroups, regardless of LVEF, sex, age, renal function, and etiology of HF [24]. The pleiotropic effects of novel HF drugs, which include neurohormonal inhibition, ventricular reverse remodeling, and upstream modulation of atrial rhythm control pathways, may improve the prognosis of patients with HF [25,26]. As is well known, in addition to the key treatment of HF, patient prognosis is also influenced by the appropriate management of comorbid conditions and the necessary use of relevant medications, such as antiplatelet drugs, statins, and hypoglycemic agents [27].

Interestingly, in the present study, OMT compliance was observed in a similar proportion of patients with HFrEF, HFmrEF, and HFpEF, but a statistically significant impact of OMT on prognosis was noted only in patients with HFrEF. OMT compliance in patients with HFrEF was associated with a significant 38% reduction in the risk of all-cause mortality after adjustment. No such association was observed for patients with HFpEF and HFmrEF.

Our results have several clinical implications. First, consistent with previous analyses, our findings indicate that patients with HF and AF may benefit from a holistic approach. Second, as demonstrated in other studies, integrated management in HF and AF patients remains suboptimal due to the insufficient implementation of guidelines in all patients. Lack of guideline adherence is more pronounced in the ABC pathway compared with that in OMT compliance. It should be noted that the lack of OMT compliance in patients with AF and HF is comparable to nonadherence to the ABC pathway. On the other hand, it should be recognized that each therapy has its limitations, such as contraindications and side effects, which may prevent its use in all eligible patients. It should also be emphasized that implementing OMT and ABC in real-world settings involves practical challenges.

## 5. Strengths and Limitations

This study is subject to several important limitations. Most stem from the nature of the data source, as the analysis was based on registry data. First, some patient records contained incomplete information. Second, the study focused exclusively on a single outcome measure, namely all-cause mortality. Third, when assessing adherence to OMT, only the presence of at least one medication from each recommended drug class was evaluated, without consideration of the actual dosages administered. Additionally, potential contraindications to specific therapies were not accounted for in the analysis. Furthermore, the recently published 2024 ESC Guidelines for the management of AF introduced the AF-CARE pathway as a comprehensive, integrated care model for patients with AF [28]. AF-CARE expands upon previous ESC recommendations, such as the five-step, outcome-oriented integrated strategy outlined in the 2016 ESC Guidelines [29] and the ABC pathway from the 2020 ESC Guidelines for AF diagnosis and management. The transition to the AF-CARE framework reflects ongoing advancements in treatment approaches and technology, particularly in rhythm control, with accumulating evidence indicating that the comprehensive management of comorbidities and risk factors enhances all aspects of AF care [30]. Lastly, the exclusion of patients who died during their index hospitalization may have led to an underestimation of overall mortality, particularly among those with acute or decompensated heart failure. This could introduce selection bias by favoring survivors who tolerated treatments, although group assignments were based on adherence criteria, with no systematic differences in treatments at discharge, thereby minimizing differential treatment bias and preserving the validity of between-group comparisons post-discharge. Nevertheless, it is important to note that the HEROES cohort is representative of the broader Polish HF population, making these findings particularly valuable and unique at the national level.

## 6. Conclusions

The present national, multicenter study provides a description of the real-life implementation of the ABC pathway and OMT compliance in patients with AF and HF. Proper implementation of the guidelines was observed in half of the study group. This result is not satisfactory, and a thorough assessment of the causes of this is needed. We found that OMT compliance can mitigate the risk of all-cause mortality in patients with HFrEF, while an integrated approach with the ABC pathway and OMT compliance reduces the risk of all-cause death. Therefore, a holistic approach to AF and HF management is strongly recommended.

## Figures and Tables

**Table 1:** Baseline characteristics and comparison of patients regarding ABC pathway and optimal medical therapy adherence.

Characteristic	All Patients N = 627	ABC + OMT Group N = 312	noABC + OMT Group N = 158	noABC + noOMT Group N = 157	*p*
Demographics					
Age, median (IQR), years	71.6 (63.8–79.1)	69.8 (62.7–76.3)	72.2 (64.3–79.8)	75.0 (65.6–80.9)	<0.001
Age group, n (%)					
<65 years	169 (27.0)	94 (30.1)	40 (25.3)	35 (22.3)	0.04
65–74 years	197 (31.4)	104 (33.3)	52 (32.9)	41 (26.1)	0.17
>74 years	261 (41.6)	114 (36.5)	66 (41.8)	81 (51.6)	0.008
Female, n (%)	206 (32.9)	95 (30.4)	51 (32.3)	60 (38.2)	0.24
Physical parameters					
BMI, median (IQR), kg/m^2^	28.4 (25.4–32.3) N = 625	29.1 (25.8–32.4) N = 311	29.1 (24.9–33.2) N = 158	27.1 (24.5–30.7) N = 156	0.007
HR, median (IQR), beats/min	72 (65–80) N = 539	72 (65–80) N = 256	70 (65–80) N = 145	74.5 (65.3–81.8) N = 138	0.13
SBP, median (IQR), mmHg	120 (106–130) N = 539	118 (105–128) N = 256	123 (110–140) N = 145	115.5 (105–130) N = 138	<0.001
DBP, median (IQR), mmHg	71 (65–80) N = 539	70 (65–80) N = 256	75 (68–80) N = 145	70 (60.5–79) N = 138	0.006
Atrial fibrillation characteristics					
Form, n (%)					
Paroxysmal	255 (40.7)	136 (43.6)	66 (41.8)	53 (33.8)	0.12
Non-paroxysmal	372 (59.3)	176 (56.4)	92 (58.2)	104 (66.2)	
CHA_2_DS_2_-VASc, median (IQR)	4 (3–5)	4 (3–5)	4.5 (3–6)	5 (3–5)	0.07
HFpEF, n (%)	173 (27.6)	67 (21.5)	57 (36.1)	49 (31.2)	0.002
HFmrEF, n (%)	130 (20.7)	69 (22.1)	33 (20.9)	28 (17.8)	0.56
HFrEF, n (%)	324 (51.7)	176 (56.4)	68 (43.0)	80 (51.0)	0.02
HF duration, median (IQR), years	3.8 (0.7–10.7)	3.6 (0.8–11.8)	4.4 (0.7–9.9)	4.1 (0.7–8.9)	0.75
HF duration, n (%)					
<1 year	182 (29.0)	89 (28.5)	47 (29.7)	46 (29.3)	0.66
1–3 years	99 (15.8)	56 (17.9)	20 (12.7)	23 (14.6)	
>3 years	346 (55.2)	167 (53.5)	91 (57.6)	88 (56.1)	
Type of visit, n (%)					
Hospitalization	539 (86.0)	256 (82.1)	145 (91.8)	138 (87.9)	0.01
Outpatient	88 (14.0)	56 (17.9)	13 (8.2)	19 (12.1)	
NYHA class, n (%) N = 539					
I	61 (11.3)	32 (12.5)	12 (8.3)	17 (12.3)	0.40
II	364 (67.5)	224 (87.5)	61 (42.1)	79 (57.3)	<0.001
III	106 (19.7)	0 (0)	66 (45.5)	40 (29.0)	<0.001
IV	8 (1.5)	0 (0)	6 (4.1)	2 (1.4)	0.004
Medical history, n (%)					
Hypertension	446 (71.1)	228 (73.1)	117 (74.1)	101 (64.3)	0.09
Diabetes mellitus	251 (40.0)	120 (38.5)	72 (45.6)	59 (37.6)	0.26
Stroke or TIA	67 (10.7)	27 (8.7)	23 (14.6)	17 (10.8)	0.15
Myocardial infarction	210 (33.5)	108 (34.6)	59 (37.3)	43 (27.4)	0.15
Stable angina	200 (31.9)	98 (31.4)	53 (33.5)	49 (31.2)	0.88
Previous VTE	19 (3.0)	11 (3.5)	7 (4.4)	1 (0.6)	0.08
Peripheral arterial disease	9 (1.4)	5 (1.6)	4 (2.5)	0 (0.0)	0.14
Chronic kidney disease	210 (33.5)	83 (26.6)	55 (34.8)	72 (45.9)	<0.001
Alcohol use, n (%)					
Current	25 (4.0)	19 (6.1)	0 (0)	6 (3.8)	0.006
Former	68 (10.8)	45 (14.4)	9 (5.7)	14 (8.9)	0.01
Never	534 (85.2)	248 (79.5)	149 (94.3)	137 (87.3)	<0.001

Data are presented as number (percentage) unless otherwise indicated. Abbreviations: ABC—Atrial Fibrillation Better Care; BMI—body mass index; DBP—diastolic blood pressure; HFmrEF—Heart Failure with Mid-Range Ejection Fraction; HFpEF—Heart Failure with Preserved Ejection Fraction; HR—heart rate; HFrEF—Heart Failure with Reduced Ejection Fraction; IQR—interquartile range; NYHA—New York Heart Association; OMT—optimal medical therapy; SBP—systolic blood pressure; TIA—transient ischemic attack; VTE—venous thromboembolism.

**Table 2:** Management strategies in the Atrial Fibrillation Better Care pathway according to optimal medical treatment for heart failure.

	All N = 627	OMT Compliance N = 470	OMT Non-Compliance N = 315	*p*
A criterion	557 (88.8)	434 (92.3)	123 (78.3)	<0.001
OAC	557 (88.8)	434 (92.3)	123 (78.3)	<0.001
VKA	113 (18.0)	83 (17.7)	30 (19.1)	0.68
DOAC	444 (70.8)	351 (74.7)	93 (59.2)	<0.001
B criterion *	513 (81.8)	398 (84.7)	115 (73.2)	0.001
Beta-blocker	583 (92.9)	457 (97.2)	126 (80.3)	<0.001
Digoxin	99 (15.8)	74 (15.7)	25 (15.9)	0.96
Amiodaron	111 (17.7)	85 (18.1)	26 (16.6)	0.66
Propafenone	5 (0.8)	3 (0.6)	2 (1.3)	0.60
AF catheter ablation	56 (8.9)	43 (9.1)	13 (8.3)	0.74
C criterion	387 (61.7)	387 (82.3)	0	<0.001
Optimal treatment of				
Hypertension	388/446 (87.0)	300/345 (86.9)	88/101 (87.1)	0.96
CAD	230/296 (77.7)	202/224 (90.2)	28/72 (38.9)	<0.001
Stroke/TIA	48/67 (71.6)	40/50 (80.0)	8/17 (47.1)	0.014
Diabetes mellitus	232/251 (92.4)	180/192 (93.8)	52/59 (88.1)	0.16

* no patients treated with diltiazem, dronedarone, flecainide, sotalol, or verapamil were recorded. Data are presented as number (percentage). Abbreviations: AF—atrial fibrillation; CAD—coronary artery disease; DOAC—direct oral anticoagulant; OAC—oral anticoagulant; OMT—optimal medical therapy; VKA—vitamin K antagonist; TIA—transient ischemic attack.

## Data Availability

The data presented in this study are openly available in [https://heroes.umed.pl, accessed on 3 November 2025] at [DOI: 10.60941/JVH1-5190].

## Supplementary Materials

The following supporting information can be downloaded at: https://www.mdpi.com/article/10.3390/jcm14238338/s1, Table S1: Multivariable Cox Regression Analyses for All-Cause Mortality in Patients with Heart Failure and Atrial Fibrillation: Associations with Heart Failure Subtypes, Left Ventricular Ejection Fraction Categories, Optimal Medical Therapy Compliance, Atrial Fibrillation Better Care Pathway Adherence, and Combined Strategies.
