# Supplementary material for: Integrated Care in Patients with Atrial Fibrillation and Optimal Medical Treatment for Heart Failure: Results from the Heart failuRe ObsErvational Study (HEROES)
Source: J Clin Med. 2025 Nov 24;14(23):8338. doi: 10.3390/jcm14238338 (PMC12692776; doi:10.3390/jcm14238338)
Supplement: Supplementary file 1 [file jcm-14-08338-s001.zip › jcm-3998024-supplementary.pdf]

Table S1. Multivariable Cox Regression Analyses for All-Cause Mortality in Patients with Heart Failure and Atrial Fibrillation: Associations with Heart Failure Subtypes, Left Ventricular Ejection Fraction Categories, Optimal Medical Therapy Compliance, Atrial Fibrillation Better Care Pathway Adherence, and Combined Strategies

Model/Category	Subgroup/Compliance	HR	95% CI	p-value
Heart Failure Subtypes				
	HFpEF (Reference)	-	-	-
	HFmrEF	1.17	0.68–2.01	0.57
	HFrEF	1.29	0.80–2.09	0.30
Left Ventricular Ejection Fraction Categories				
	LVEF 10–19% (Reference)	1.00	-	-
	LVEF 20–29%	0.86	0.44–1.66	0.65
	LVEF 30–39%	1.07	0.57–2.03	0.83
	LVEF 40–49%	0.88	0.45–1.73	0.72
	LVEF 50–59%	0.79	0.39–1.59	0.50
	LVEF ≥60%	0.69	0.29–1.61	0.39
Optimal Medical Therapy (OMT) Compliance				
	No OMT (Reference)	-	-	-
	OMT	0.61	0.42–0.90	**0.01**
HF Subgroups Stratified by OMT				
	HFpEF (Reference)	-	-	-
	HFmrEF, OMT	1.05	0.57–1.93	0.87
	HFmrEF, no OMT	1.52	0.66–3.49	0.32
	HFrEF, OMT	1.01	0.60–1.72	0.97
	HFrEF, no OMT	1.95	1.11–3.42	**0.02**
HF Subgroups with OMT (No OMT as Reference)				
	No OMT (Reference)	-	-	-
	HFpEF, OMT	0.58	0.34–1.00	**0.05**
	HFmrEF, OMT	0.65	0.36–1.15	0.14
	HFrEF, OMT	0.62	0.39–0.98	**0.04**
Atrial Fibrillation Better Care (ABC) Pathway Adherence				
	No ABC (Reference)	-	-	-
	ABC	0.74	0.51–1.09	0.12
HF Subgroups Stratified by ABC				
	HFpEF (Reference)	-	-	-
	HFmrEF, ABC	1.37	0.73–2.58	0.32
	HFmrEF, no ABC	0.93	0.44–1.96	0.85
	HFrEF, ABC	0.96	0.54–1.72	0.89
	HFrEF, no ABC	1.62	0.97–2.73	0.07

Model/Category	Subgroup/Compliance	HR	95% CI	p-value
HF Subgroups with ABC (No ABC as Reference)				
	No ABC (Reference)	-	-	-
	HFpEF, ABC	0.55	0.26–1.17	0.12
	HFmrEF, ABC	1.04	0.59–1.83	0.90
	HFrEF, ABC	0.70	0.43–1.13	0.15
Combined Compliance				
	No ABC + No OMT (Reference)	-	-	-
	ABC + OMT	0.60	0.39–0.92	**0.02**
	No ABC + OMT	0.64	0.40–1.02	0.06

Notes: All models were adjusted for age, sex, body mass index, hypertension, and diabetes mellitus as potential confounders, selected due to their known impact on mortality in heart failure and atrial fibrillation populations. Abbreviations: ABC, Atrial Fibrillation Better Care pathway; CI, confidence interval; HF, heart failure; HFmrEF, Heart Failure with Mid-Range Ejection Fraction; HFpEF, Heart Failure with Preserved Ejection Fraction; HFrEF, Heart Failure with Reduced Ejection Fraction; HR, hazard ratio; LVEF, left ventricular ejection fraction; OMT, optimal medical therapy.
